# Half-dose photodynamic therapy versus 577 nm subthreshold pulse laser therapy in treatment-naive patients with central serous chorioretinopathy

**DOI:** 10.1186/s12886-023-03274-9

**Published:** 2024-01-04

**Authors:** Vasilena Sitnilska, Petra Schiller, Tim U. Krohne, Lebriz Altay

**Affiliations:** 1 Department of Ophthalmology, Faculty of Medicine, University Hospital Cologne, University of Cologne, Cologne, Germany; 2 Institute of Medical Statistics and Computational Biology (IMSB), Faculty of Medicine, University of Cologne, Cologne, Germany

**Keywords:** Central serous chorioretinopathy, Half-dose photodynamic therapy, Prognostic factors, Subliminal laser

## Abstract

**Background:**

To compare real-life anatomical and functional outcomes of half-dose photodynamic therapy (HD-PDT) and 577 nm subthreshold pulse laser therapy (SPL) in treatment-naïve patients with central serous chorioretinopathy (CSC).

**Methods:**

We retrospectively reviewed consecutive treatment-naïve CSC patients with non-resolving subretinal fluid (SRF) for more than 2 months who received either HD-PDT or SPL treatment. One repetition of the same treatment was allowed in patients with persistent SRF after first treatment. Functional and anatomical outcomes were assessed after first treatment and at final visit.

**Results:**

We included 95 patients (HD-PDT group, n = 49; SPL group, n = 46). Complete resolution of SRF after a single treatment was observed in 42.9% of HD-PDT-treated patients (n = 21; median time to resolution 7.1 weeks) and in 41.3% of SPL-treated patients (n = 19; median time to resolution 7.0 weeks). In the HD-PDT-group, 44.9% of patients (n = 22) and in the SPL-group, 43.5% (n = 20) of patients, received a second treatment due to persistent SRF, while 12.2% (n = 6) and 15.2% (n = 7), respectively, opted against a second treatment despite persistent SRF. After the final treatment, complete SRF resolution was observed in 61.2% of all HD-PDT-treated patients (n = 30; median time to resolution 8.8 weeks) and 60.9% of all SPL-treated patients (n = 28; median time to resolution 13.7 weeks, *p* = 0.876). In the final visit, both groups showed significant improvement of BCVA in comparison to baseline (*p* < 0.001 for all). The change in BCVA from baseline to final visit was similar for the two groups (HD-PDT, median BCVA change 0.10 logMAR (IQR: 0.0-0.2); in SPL group, median BCVA change 0.10 logMAR (IQR: 0.0-0.2), *P* = 0.344). The CSC subclassification (simple versus complex) had no influence on the anatomical or functional outcome.

**Conclusions:**

High-density 577 nm SPL resulted in as good anatomical and functional treatment as HD-PDT and may thus represent a treatment alternative to HD-PDT in CSC.

## Background

Central serous chorioretinopathy (CSC) is a posterior segment disease affecting the neurosensory retina. It causes an accumulation of subretinal fluid (SRF) and alteration in the retinal pigment epithelium (RPE) due to choroidal disturbances [1]. These morphological changes lead to blurred or decreased vision, which occurs mostly in middle aged men [1]. In most cases a self-limiting absorption of SRF leads to restoration of the central vision. Yet, in estimated 10–20% of the cases the disease takes a more chronical course with persisting SRF and permanent structural damage of the RPE [2]. If left untreated, chronic CSC may lead to irreversible vision decline with concomitant decrease in quality of life [3].

Over the years, various treatment options have been proposed for chronic CSC including conventional laser photocoagulation, transpupillary thermal therapy and oral therapy with mineralocorticoid receptor antagonist; partly despite their possible side effects [4–10]. Data from randomized controlled trials (RCTs) supports the efficacy and safety of photodynamic therapy (PDT) in the management of chronic CSC [11]. Moreover, half-fluence or half-dose PDT (HD-PDT) has been reported to demonstrate similar effectiveness to full-dose PDT [12–14]. However, a HD-PDT treatment requires intravenous injection of photosensitive medication verteporfin, longer time to perform, as well as higher costs of the drug. Additionally, there is currently a world-wide shortage of visudyne. Furthermore, contraindications to verteporfin infusion include pregnancy, porphyria, poor liver function and high systemic photosensitivity (48 h post treatment) and verteporfin adverse effects such as dizziness and back pain have been reported [15].

As an alternative or complementary treatment modality, several groups reported encouraging results after treatment with high-density subthreshold pulse laser treatment (SPL) modalities of various wavelengths [9, 10, 15–23]. Recent evidence highlighted the HD-PDT to be superior in comparison to 810 nm SPL [11]. Yet other studies employing 577 nm laser wavelength with individual power titration found SLP to be as efficacious as HD-PDT [15, 17].

The aim of this study was to compare effectiveness of 577 nm high-density SPL with power titration to HD-PDT in real-life settings. In contrast to our previous report, in which we have investigated this approach in a mixed cohort of pre-treated and treatment-naïve patients [17], we included in this study only treatment-naïve CSC patients with persisting complaints more than two months.

## Methods

### Patients

This retrospective study reviewed all available patients’ charts with diagnosis of CSC between January 2012 and February 2018 in Department of Ophthalmology, University clinic of Cologne (n = 328). From those 328 assessed cases, 88 patients did not receive any therapy, 25 patients showed other concomitant retinal diseases (such as drusen, high myopia, pseudovitelliform lesion, uveitis etc.), 49 patients were not therapy-naïve and 32 patients were excluded due to lack of retinal imaging 4–10 weeks after first treatment. Also patients with signs of macular neovascularization (MNV) (n = 16), signs of polypoidal vasculopathy (n = 2), patients without subfoveal SRF (n = 1) and patients with intraretinal cysts as possible degenerative sign (n = 5) were excluded. Further fifteen patients were excluded due to change in treatment modalities after the first cycle. None of the included patients had signs of other retinal diseases (myopia > 6dpt, age-related macular degeneration, macular dystrophy, diabetic retinopathy). All included patients had visual complaints on the affected eye for more than 2 months. Patient charts were reviewed for demographic data and medical history (presence of hypertension/systemic or local steroid use). Best-corrected visual acuity (BCVA) in logarithm of the minimum angle of resolution (logMAR) was noted at baseline and at the end of the study for all patients. The study was in accordance with the tenets of the Declaration of Helsinki and the Medical Research Involving Human Subjects Act (WMO).

### Diagnosis and grading of CSC

Diagnosis and grading of all patients was performed using the nomenclature system suggested recently by the Central Serous Chorioretinopathy International Group [24]. Briefly, CSC was diagnosed when presence of RPE alterations identified on fundus autofluorescence (FAF) imaging and presence or evidence of prior SRF was seen in SD-OCT, infrared imaging or fundus autofluorescence, Additionally, one of the following was also present: hyperfluorescent areas on indocyanine green angiography (ICGA), focal leaks on fluorescein angiography (FA) or more than 400 μm choroidal thickness. Choroidal neovascularization (CNV) was defined by a late leakage on FA, corresponding to a late staining area on ICGA and flat irregular, hyperreflective RPE elevation on SD-OCT [25]. The grading was performed on FA/ICGA (Spectralis HRA + OCT; Heidelberg Engineering, Heidelberg, Germany), FAF and SD-OCT (scan area 20° x 15° (5.9 × 4.4 mm), 37 B-scans, distance between B-scans 123 mm) by two independent graders (VS, LA).

All CSC patients were classified as either simple or complex according to the total area of RPE alteration identified on FAF imaging as follows: simple CSC had total area of RPE alterations ≤ 2 Disc area (DA) and complex had total area of RPE alteration > 2 DA or multifocal area. In all cases, patients’ visual complaints had been lasting longer than 2 months.

Further additional morphological features baseline were included: presence of hotspot on ICGA, central retinal thickness (CRT) and subfoveal SRF /Photoreceptors (PR) thickness (1 mm diameter grid centered on the fovea). For all measurements, automated segmentation of Heidelberg Eye Explorer software was used and all results were corrected manually. Hereby CRT was measured from internal limiting membrane to Bruch´s membrane (BrM), SRF/PR thickness was measured as the distance between external limiting membrane and BrM. Further, presence of active or inactive CSC at the fellow eye at baseline was graded. All baseline characteristics are summarized in Table 1.

**Table 1:** Demographics

	HD-PDT	SPL	*P*-Value
Number of patients, n (%)	49 (51.6)	46 (48.4)	
Age (years), median (IQR)	51.0 (45.5–57.5)	47 (42.3–55.0)	0.119
Male sex, n (%)	32/49 (65.3)	36/46 (78.3)	0.162
Disease duration (years), median (IQR)	0.59 (0.32–1.56)	0.96 (0.35–3.09)	0.288
Complex CSC subclassification, n (%)	25 (51.0)	28 (60.9)	0.334
Steroid use within last 3 months, n (%)	5/46 (10.9)^*^	7/40 (17.5)^*^	0.376
Affected fellow eye, n (%)	25/49 (51.0)	27/46 (58.7)	0.453
Hypertension, n (%)	10/49 (20.4)	13/46 (28.3)	0.372
Hotspot on ICGA, n (%)	27/44 (61.4)^†^	34/43 (79.1)^†^	0.071
BCVA (logMAR), median (IQR)	0.2 (0.2–0.4)	0.2 (0.2–0.4)	0.527
CRT (µm), mean ± SD	416.1 ± 122.9	423.2 ± 117.4	0.445
SRF/PR (µm), mean ± SD	256.5 ± 113.3	256.7 ± 114.0	0.973

Legend: Pearson’s chi² test for categorical variables; Mann-Whitney U Test for continuous variables. *Missing data Steroid use within 3 months: 3 cases in half dose PDT group, 6 cases in SPL group. †Missing data Hotspot on ICG-A: 5 cases in half dose PDT group, 3 cases in SPL group. BCVA: Best corrected visual acuity, CRT: Central retinal thickness, ICGA: Indocyanine green angiography, IQR: Interquartilrange (IQ percentile 25%- IQ percentile 75%), HD-PDT: Half-Dose photodynamic therapy, OD: Optic disc diameter, PR: Photoreceptors, SD: Standard deviation, SPL: Subthreshold pulse laser, SRF: Subretinal fluid

### Patient treatment

**Fig. 1:**
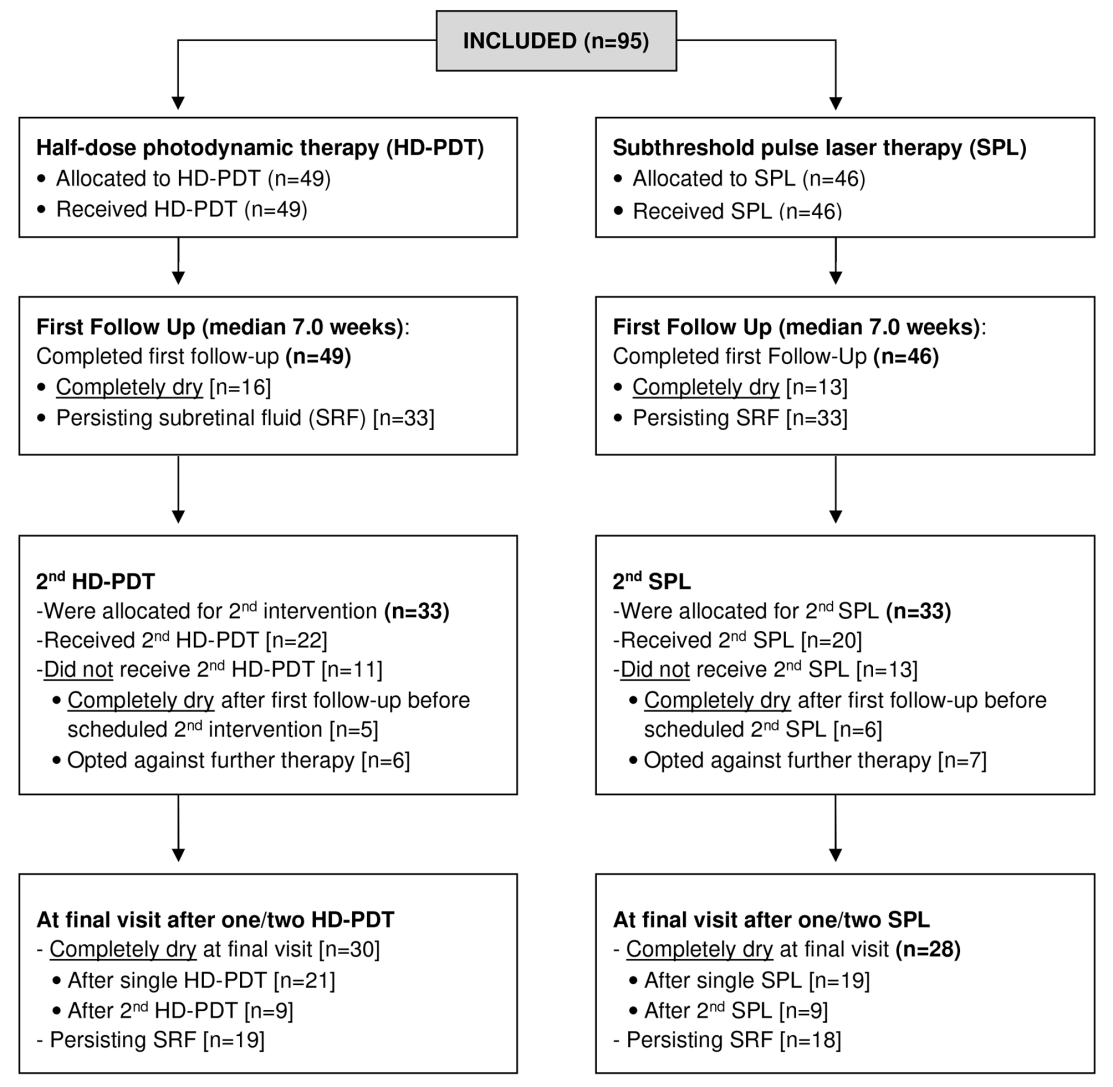
Flow chart

All included patients underwent treatment with either HD-PDT or SPL in the Department of Ophthalmology, University of Cologne (Fig. 1). Before making therapy decision, all patients were informed about both treatment modalities. Physicians and patients together decided which therapy should be initiated as first-line based of patient preferences and location of the CSC lesion. As transfoveal SPL treatment was not allowed within this study, in cases of hyperpermeability of choroidal vessels within 500 μm radius of the fovea HD-PDT treatment was advised.

**Fig. 2:**
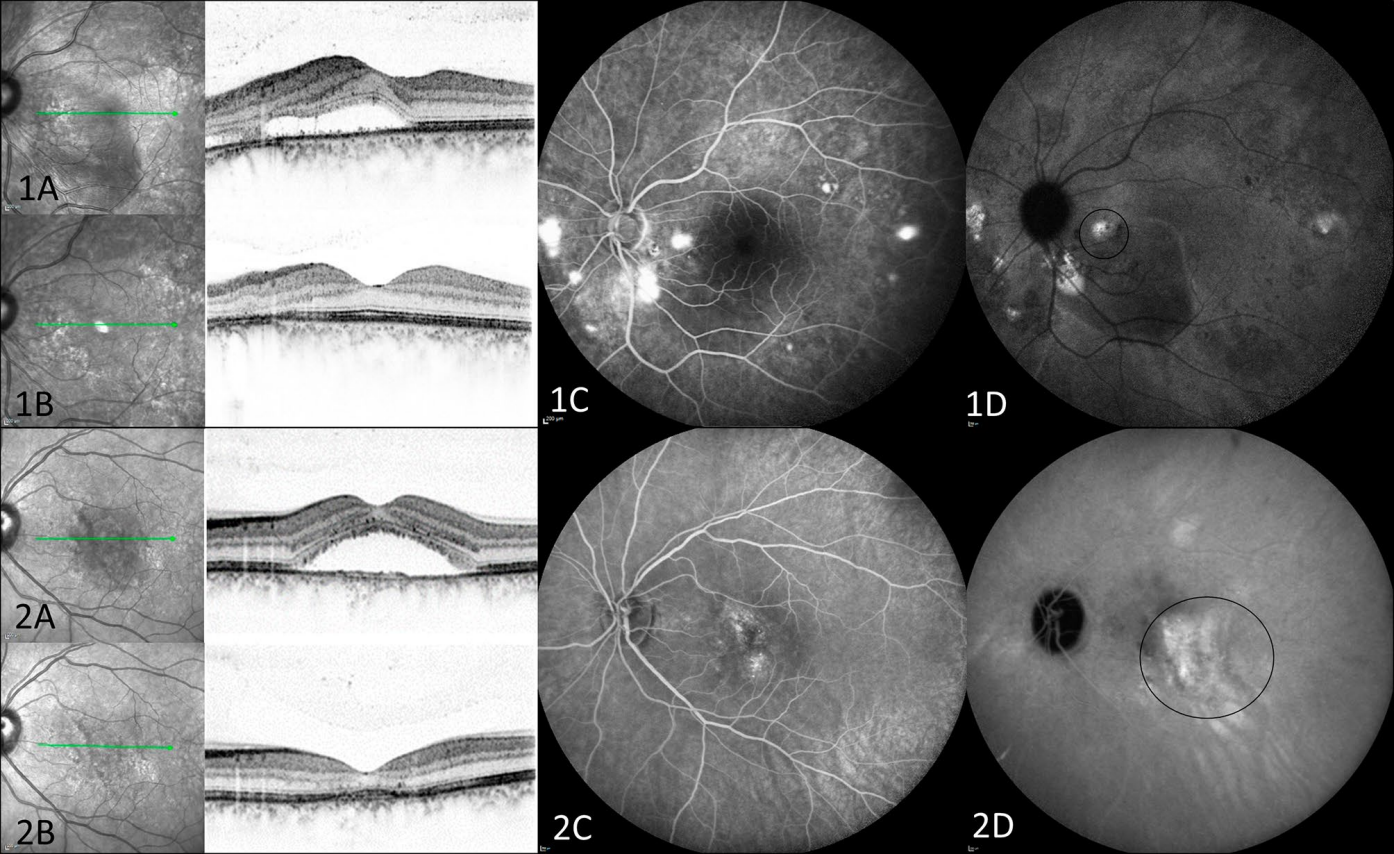
Patients examples with selection of treatment areas. **1A)** and **2A)** Baseline optical coherence tomography (OCT), **1 C)** and **2 C)** Fluorescein angiography shows hyperfluorescent areas, **1D)** and **2D)** Treatment area according to mid-phase indocyaningreen angiography. **1B)** OCT finding after treatment with subthreshold pulse laser, **2B)** OCT finding after treatment with half-dose photodynamic therapy

Treatment with HD-PDT or SPL was applied on hyperfluorescent spots on mid-phase ICGA and the corresponding “hotspots” and leakage areas on mid-phase FA (Fig. 2). Both treatment modules were applied using Area Centralis contact lens (laser spot magnification x0.94). For HD-PDT, first an intravenous infusion with verteporfin (3mg/m^2^, Visudyne®) was injected with consecutive treatment: 50 J/ cm^2^ fluency, 689 nm laser wavelength and 83s duration on previously defined area [17].

Following standardized parameters were used for SPL: 130 μm spot size, 0.2s exposure time, 5% duty cycle (Supra Scan 577 nm laser, Quantel Medical, Cedex, France). Power was titrated for each individual initially outside the macula until the laser effect was barely visible (threshold power). As next step, the energy was reduced 50% of the chosen threshold power and was applied on the previously defined area. Dense multispot delivery (9 spots grid) without any spacing between spots was applied to ensure the homogenous applications of the subthreshold laser spots.

All included patients had an ocular medical examination in 4 to 10 weeks after first treatment and were assigned to a second treatment if needed according to clinician decision (Fig. 1). In cases of complete SRF resolution after 4–10 weeks (‘’completely dry’’), no second treatment was applied. In cases with persisting SRF further controls with optional second treatment was scheduled. In cases where there was a significant but not complete resolution of SRF, a decision regarding further treatment was taken individually according to clinical improvement of visual acuity, patient complaints and outcome in further control appointments. In cases with persisting SRF, a second cycle of treatment was applied. Patients who showed persisting SRF on SD-OCT even after the second treatment were categorized as ‘’non-responders’’. Patients who received a second treatment different from the first one (therapy switch) were excluded from this study. Patients who did not appear for their second treatment were considered as lost to follow-up. Therapy success was defined as complete SRF resolution (‘’completely dry’’).

### Statistical analyses

Descriptive statistics were used to summarize the characteristics of the two groups of patients. Results were given as mean ± standard deviation, median ± interquartile range (IQR) or number of patients and percentage. Pearson’s chi² test was used for categorical variables and t-test or Mann-Whitney U Test for continuous variables depending on the distribution. Change in BCVA between the groups was compared using Mann Whitney U Test as BCVA and the change in each group was not normally distributed.Further potential prognostic factors for treatment outcome (age/ gender/ therapy/ steroid use/ affected fellow eye/ hypertension/ BCVA/ presence of hotspot/ CRT/ SRF-PR thickness) were analyzed as single factors using logistic regression analysis. Statistical analyses were performed using SPSS (IBM SPSS Statistics for Windows, Version 25.0. Armonk, NY: IBM Corporation). P values < 0.05 were considered as statistically significant.

## Results

From 328 subjects assessed for eligibility, 95 met all inclusion and none of the exclusion criteria. According to the applied nomenclature system [24], 42 (44.2%) of the patients were classified as simple and 53 (55.8%) were classified as complex CSC. There was no significant difference in the CSC subclassification distribution between patients who received HD-PDT and those who received SPL (*p* = 0.334). Baseline characteristics of included patients were comparable in both groups **(**Table 1**).** No recorded adverse events were found in the patients’ files after the application of HD-PDT or SPL.

### Anatomical outcome

**Fig. 3:**
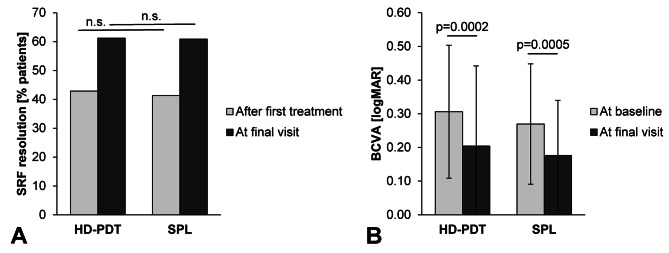
**(A)** Anatomical outcome, **(B)** Functional outcome after first treatment and at the final visit. Abbreviations: BCVA: Best corrected visual acuity, HD-PDT: half-dose photodynamic therapy, SPL: subliminal laser, SRF: Subretinal fluid

After the final treatment, 61.2% of the HD-PDT group (30 of 49 patients) and 60.9% of SPL group (28 of 46 patients) were completely dry (*p* = 0.972; Table 2; shown in Fig. 3). The median time until reaching a completely dry status overall was 8.8 (IQR 7.0-20.4) and 13.7 (IQR 6.4–23.7) weeks, respectively (*P* = 0.876). Hereby, 42.9% of HD-PDT group (n = 21) and 41.3% of SPL (n = 19) were completely dry after single treatment cycle. The median complete SRF resolution time was 7.1 (IQR: 6.7–11.5) and 7.0 (IQR: 6.0–19.0) weeks after a single treatment in the HD-PDT and the SPL group (*P* = 0.789). The median observation time was 7.2 (IQR: 6.64–8.64) and 8.2 (IQR: 6.3–12.3) weeks after a single treatment in the HD-PDT and the SPL groups (*P* = 0.276).

The classification into simple or complex disease did not have any effect on the overall treatment success. Patients with simple CSC subclassification achieved a completely dry status within a median duration of 7.14 weeks, while those categorized as complex CSC subclassification required a significantly longer median time of 15.7 weeks (*p* = 0.010).

**Table 2:** Final analysis: HD-PDT versus SPL after one/two treatment cycles

	HD-PDT (n = 49)	SLL (n = 46)	*P*-Value
Total observation time (weeks, median(IQR))	17.3 (9.9–24.2)	17.7 (9.0-25.5)	0.908*
Patients with second treatment (n,%)	22 (44.9)	20 (43.5)	0.889†
Change in BCVA (logMAR, median (IQR))	0.10 (0.0-0.2)	0.10 (0.0-0.2)	0.344*
Completely dry (n,%)	30 (61.2)	28 (60.9)	0.972†

Legends: * Mann-Whitney U Test, † Pearson’s chi² test. BCVA: Best corrected visual acuity, 95%CI: Confidence interval, IQR: Interquartile range, logMAR: Logarithm of the minimum angle of resolution, HD-PDT: Half-Dose photodynamic therapy, SD: Standard deviation, SPL: Subthreshold pulse laser

### HD-PDT group

At first follow-up (median observation time 7,0 weeks after first treatment), 16 of 49 patients (32.7%) showed complete resolution of SRF, while 33 patients (67.3%) had persistent SRF (Fig. 1). Before the initiation of the second HD-PDT therapy, 5 more patients showed complete SRF resolution and therefore did not require a second treatment. Of the remaining 28 patients with persistent SRF, 6 opted against further therapy and 22 received a second HD-PDT treatment. Median time between the first and the second treatment for those 22 patients was 14.00 weeks (IQR: 9.57–21.50). Of the 22 patients with a second treatment, 9 were completely dry afterwards, 13 had persistent SRF (Fig. 1). Thus, a total of 30 (61.2%) of all 49 HD-PDT-treated patients demonstrated complete SRF resolution (Fig. 1).

The patients with simple CSC sub classification were not associated with higher odds of achieving a completely dry status after HD-PDT compared to individuals with the complex CSC subclassification. (*p* = 0.684, Odds Ratio (OR): 0.79, *p* = 0.684, 95% Confidence interval (CI): 0.249–2.490). Time from the first HD-PDT treatment to completely dry was median 7.92 weeks in patients with simple CSC subclassification, and 13.71 weeks in patients with complex CSC (*p* = 0.313).

### SPL group

Thirteen of 46 SPL-treated patients (28.3%) were completely dry at the first follow-up (median observation time 7,0 weeks after first treatment), 33 (71.7%) had persisting SRF. Before scheduled second SPL treatment, 6 more patients showed complete SRF resolution and did therefore not receive a second treatment. This was similar to the rate of 42.9% in the HD-PDT group (*p* = 0.878). Of the remaining 27 patients with persistent SRF, 7 chose not to receive further therapy while 20 were treated with a second SPL. Median time between the first and the second treatment for those 20 patients was 13.57 weeks (IQR: 10.21–24.14). This was similar to the median time between first and second therapy in the HD-PDT group (*p* = 0.797). Of the 20 patients with a second treatment, 9 were completely dry, 11 had persisting SRF (Fig. 1). Thus, a total of 28 (60.8%) of all 46 SPL-treated patients demonstrated complete SRF resolution (Fig. 1).

The subclassification of complex versus simple CSC had no influence on the completely dry outcome (*p* = 0.519, OR: 1.50. 95% CI: 0.437–5.148), however patients with simple CSC status had significantly shorter interval after the first SPL until completely dry status (median of 6.43 weeks) in comparison to those with complex CSC (median of 20.00 weeks, *p* = 0.015).

### Functional outcome

In the final visit, both HD-PDT and SPL groups showed a significant improvement of BCVA in comparison to baseline. In the HD-PDT group, the mean BCVA was 0.31 ± 0.20 at baseline and 0.20 ± 0.24 at the final visit (*p* < 0.001). In the SPL group the mean BCVA was 0.27 ± 0.17 at baseline and 0.17 ± 0.16 at the final visit (*p* < 0.001). In both groups, the median BCVA change was 0.1 logMAR (IQR:0.0-0.2, *P* = 0.344, shown in Fig. 3; Table 2). The CSC status of simple or complex had no influence to functional outcome. Both simple and complex CSC showed also a median BCVA change of 0,1 logMar (IQR:0.0-0.2, *P* = 0.823).

### Prognostic factors for complete SRF resolution

Younger patients showed a higher likelihood for the complete resolution of the fluid after one/two cycles of SPL (*p* = 0.031, OR: 0.92, 95% CI: 0.86–0.99). In the HD-PDT group, age showed no association with treatment response (*p* = 0.192, OR: 0.95, 95% CI: 0.88–1.03). Overall, presence of hotspot was slightly associated with a higher change to have “completely dry” status (*p* = 0.047, OR: 2.60, 95% CI 1.01–6.65), yet after adjustment for age, the association was insignificant (*p* = 0.154). The impact of hotspot on treatment response could also not be detected in each group separately (HD-PDT and SPL, *p* > 0.05). Similarly, greater baseline SRF accumulation was also slightly associated with a better treatment outcome (CRT: *p* = 0.031, OR: 1.00, 95%CI 1.00-1.01; SRF/PR: *p* = 0.014, OR: 1.01, 95%CI 1.00-1.01), yet the associations were not significant in each group subanalysis (HD-PDT and SPL, *p* > 0.05). Gender, prior or current steroid use, CSC in the fellow eye, arterial hypertension and BCVA at baseline, did not show a significant association (p all > 0.05).

## Discussion

In this retrospective study, we compared the therapeutic success of HD-PDT and SPL with up to two consecutive treatment cycles each in treatment-naïve CSC patients with non-resolving complaints for more than two months. Both treatment modalities had comparable anatomic and visual outcomes, as both showed comparable rates of reaching completely dry status (Fig. 3) and a similar BCVA increase. Our results indicate that both therapeutic interventions are successful in more than 60% of patients with regard to complete resolution of SRF in a median observation mean time of 17 weeks. In this cohort, the presence of hotspots and a greater baseline SRF accumulation were slightly associated with a better treatment outcome, which correlates with the results shown by Singh et al. [26] Nevertheless, these associations were not significant when tested in the different treatment groups as subanalyses.

In previous studies, complete SRF resorption was reported as between 67% and 100% following HD-PDT [11, 27–29] and between 29% and 100% after SPL/micropulse treatment [11, 15, 30–33]. At the final visit of this study, 61.2% of patients showed complete SRF resolution after HD-PDT therapy. This percentage is similar to the results of the prospective PLACE study, where 67.2% of the patients with HD-PDT treatment were completely dry. In contrast, in the PLACE study only 28.8% were completely dry after subthreshold 810 nm micropulse laser treatment, whereas in our cohort 60.9% were completely dry after 577 nm SPL. One possible reason for this discrepancy may be related to the usage of lasers with different wavelengths: in the PLACE study 810 nm wavelength micropulse laser was used, whereas the patients in our study were treated with a 577 nm wavelength SPL laser. Besides different wavelength compared to the PLACE study, different laser settings were used: We used multispot delivery and individual laser energy titration, whereas the PLACE study used a fixed energy without energy titration. In comparison to our previous report, [17] in which also pre-treated patients were included, the percentage of treatment response after SPL was lower in this cohort (61% in our current study versus 79% in [17]). This may be related to the stricter definition of therapy response in our current study (completely dry in contrast to the previous definition of therapy response as decrease in CRT to a minimum of 20 μm) as well as to the different patient cohort with exclusion of pre-treated patients in our current study [17].

HD-PDT is assumed to cause choriocapillary vessel remodeling inducing active repair mechanisms [34]. SPL treatment has been suggested to induce RPE remodeling without causing photoreceptors or RPE damage. The exact mechanism of SPL success remains unclear, but it is assumed that it causes upregulation of heat shock proteins that play important role in stabilizing protein structure in stress situations [35]. So far different types of laser treatment have been introduced, all with different wavelength, duty cycle and other laser settings [22, 23]. This makes it difficult to compare results of different trials and to select the best option for treatment. Although CSC is thought to be primarily a choroidopathy, the RPE probably plays an important role in resolving symptoms, and it is possible that the shorter wavelength laser light of 577 nm is more effective in affecting the RPE than 810 nm micropulse laser light. Prospective studies with a larger CSC cohort may be helpful to understand the role of different wavelengths on therapy outcome. So far, no prospective study are published comparing treatment outcomes of 577 and 810 nm SPL or of 577 nm SPL and HD-PDT in chronic CSC patients. To date, 577 nm SPL has shown good therapeutic effect without inducing any tissue damage [15–18, 21–23, 36]. Because of the fact that correct SPL application is intended to not result in a visible laser spot, there is a chance of undertreatment which may provide an additional explanation for the high variety of published treatment results [37]. No adverse effects of subthreshold pulse laser application have been reported [37, 38]. In contrast, reported adverse effects of HD-PDT include choroidal non-perfusion, RPE atrophy, secondary MNV, and transient retinal function impairment [39–41].

Micropulse laser treatment was considered to have better outcome in patients with focal leakage rather than diffuse leakage [42]. In this study, presence of a hotspot on ICGA showed a tendency towards positive impact on treatment response, however the association did not remain statistically significant after age-adjustment. These results are consistent with the PLACE study, where therapy outcome remained stable after comparing two subgroups with focal or diffuse leakage [43].

A multimodal imaging based classification system was already been shown as useful for planning further treatment for CSC [27]. In this study, both treatment modalities had a similar functional and anatomical outcome in patients with simple and complex CSC. Patients with complex CSC had a significantly longer time for SRF resolution, which was consistent with the results Arora et al [27] However, simple or complex CSC subclassification had no statistical influence on SRF resolution time if the patients were treated only with HD-PDT.

Limitations of this study include the small cohort, the retrospective nature, the non-randomized treatment options, the potential treatment bias, and the possibility of undetected MNV at baseline due to lack of OCT-A in all patients. OCT-A may have detected smaller MNV, not visible on FA [39]. In such cases, treatment with HD-PDT would be expected to result in a better outcome [40]. Furthermore, enhanced deep imaging (EDI) was not available for all patients, and therefore choroidal thickness measurements could not be performed.

## Conclusions

In conclusion, SPL shows good functional and anatomical outcomes in CSC patients comparable to HD-PDT. While HD-PDT will remain as the best established treatment for chronic CSC, our results suggest SPL as an alternative or complementary treatment especially in centers where PDT is not available due to lack of the laser device of the required drug. Furthermore, SPL can be considered as a viable therapeutic option in CSC patients with contraindications for HD-PDT.

## Data Availability

The datasets used and/or analysed during the current study are available from the corresponding author on reasonable request.

## Abbreviations

BCVA: Best-corrected visual acuity
BrM: Bruch´s membrane
CRT: central retinal thickness
CSC: central serous chorioretinopathy
DA: disc area
FA: fluorescein angiography
HD-PDT: half-dose photodynamic therapy
ICGA: indocyanine green angiography
logMAR: logarithm of the minimum angle of resolution
MNV: macular neovascularization
PR: Photoreceptors
RPE: retinal pigment epithelium
SPL: subthreshold pulse laser therapy
SRF: subretinal fluid
